# Pneumothorax Ex Vacuo: A Rare Complication of PleurX Catheter Insertion

**DOI:** 10.7759/cureus.41882

**Published:** 2023-07-14

**Authors:** Faisal Syed, Ramya Pakala, Md Didar Ul Alam, Gagan P Singh, Vishal Poddar

**Affiliations:** 1 Internal Medicine, Howard University Hospital, Washington DC, USA; 2 Pulmonary and Critical Care, Howard University College of Medicine, Washington DC, USA; 3 Pulmonary and Critical Care, Howard University Hospital, Washington DC, USA

**Keywords:** pleural effusion, pleurx catheter, trapped lung, adenocarcinoma, pneumothorax ex vacuo

## Abstract

Pneumothorax ex vacuo (PEV) is a rare type of pneumothorax that occurs when air enters the pleural space in the chest cavity due to an increase in the volume of the lungs or a reduction in the volume of the surrounding lung tissue. Unlike a typical pneumothorax, which involves the collapse of the lung due to air accumulation, pneumothorax ex vacuo occurs when the lung itself cannot expand properly, often due to underlying lung disease or conditions such as pulmonary fibrosis or atelectasis. The mechanism is compensatory to the lung entrapment. PleurX catheter (Pleur-Evac; Teleflex, Wayne, PA, USA) insertion can cause pneumothorax ex vacuo in patients with cancer histories, as shown in this case. It is important to understand if pneumothorax ex vacuo needs observation or quick intervention. Pleural manometry is also an important part of diagnosis of pneumothorax ex vacuo and we discuss that in our case report.

## Introduction

Pneumothorax ex vacuo (PEV) is an uncommon but subtle complication of non-expandable lung. In pneumothorax ex vacuo, lung is unable to re-expand due to low pleural pressure occuring from pleural effusion and drainage, creating a vacuum phenomenon [1]. Pneumothorax ex vacuo after insertion of a pleural catheter post thoracentesis is rare. We present a case of pneumothorax ex vacuo status post PleurX catheter (Pleur-Evac; Teleflex, Wayne, PA, USA) placement due to trapped lung requiring video-assisted thoracic surgery (VATS) and decortication. The aim of this case report is to identify how and when pneumothorax ex vacuo occurs, especially in patients with risk factors like malignancy. This case report also highlights the treatment options for pneumothorax ex vacuo from simple observation versus need of surgical intervention. Early diagnosis with pleural manometry can be beneficial in differentiation of benign pneumothorax ex vacuo versus more severe tension pneumothorax.

## Case presentation

**Figure 1:**
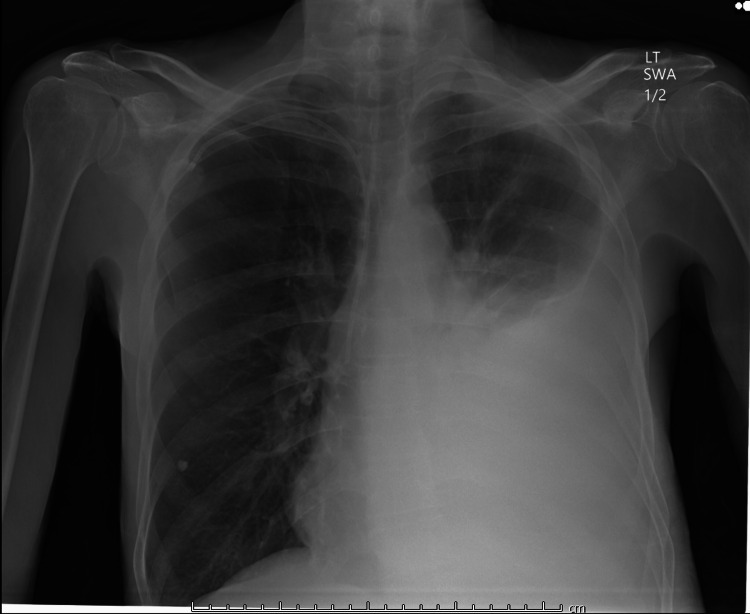
Patient's Chest X-ray two weeks ago showing left-sided pleural effusion

**Figure 2:**
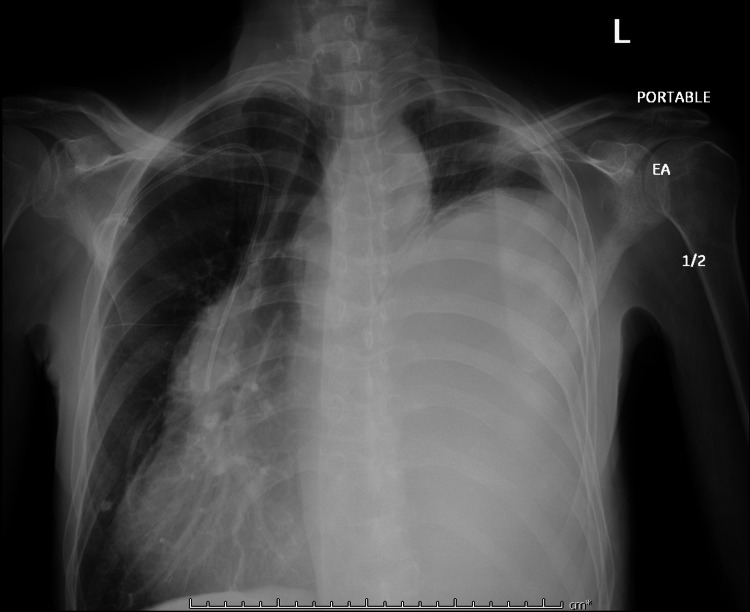
Chest X-ray on admission now showing increase in left-sided pleural effusion

A 63-year-old male presented to the ED with worsening shortness of breath and productive cough for three weeks. Patient was evaluated two weeks ago for left-sided effusion seen in chest X-ray (Figure 1). Pleural fluid cytology at that time was positive for lung adenocarcinoma. His other medical history included asthma, bronchitis, and small lymphocytic B cell lymphoma s/p chemotherapy. On arrival, vitals showed blood pressure (BP) 133/99, pulse 77, temperature 97.7, saturating 98% on room air. On physical exam, the patient had decreased breath sounds on the right lower lung field. Initial Chest X-ray showed significantly worsening large left-sided pleural effusion that had re-accumulated (Figure 2).

**Figure 3:**
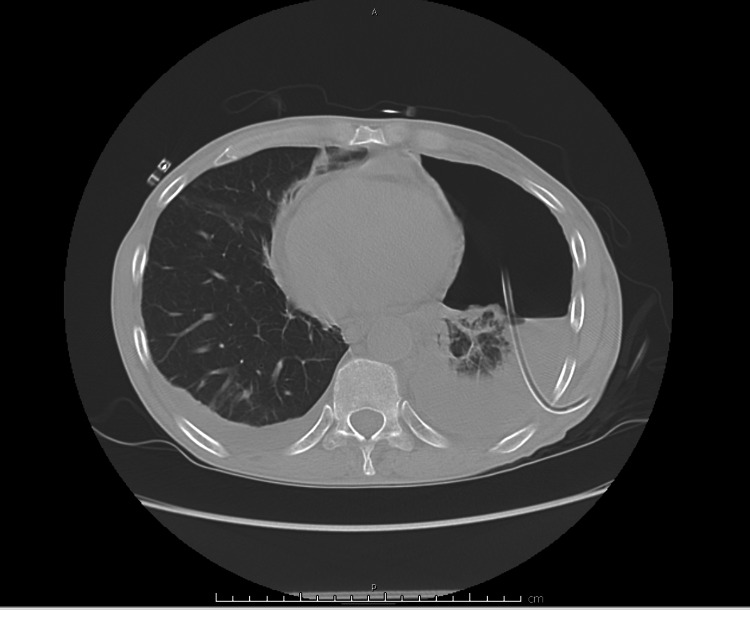
CT Chest post PleurX catheter placement

**Figure 4:**
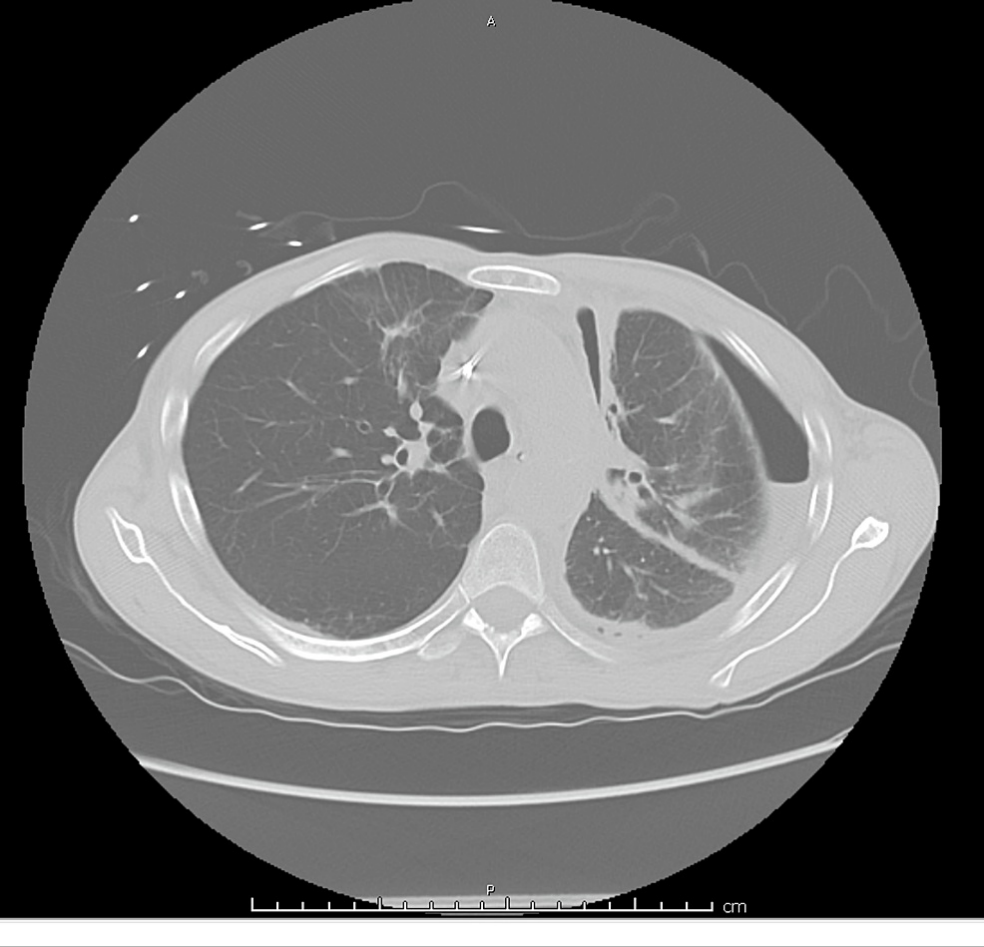
CT Chest showing entrapped lung with pneumothorax ex vacuo

Due to rapid accumulation of pleural fluid, a PleurX catheter was placed during this admission. Repeat CT chest without contrast was showing limited lung expansion. Although there were no signs of endobronchial lesion, imaging was significant for pneumothorax ex vacuo (Figure 3, 4).

**Figure 5:**
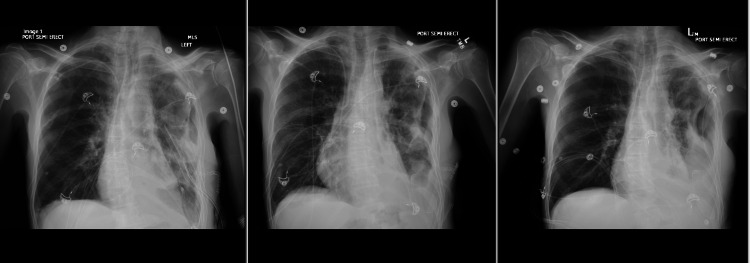
Daily Chest X-rays post video-assisted thoracic surgery (VATS) procedure

Despite the PleurX catheter placement, patient continued to have persistent pneumothorax and was symptomatic with respiratory distress. Due to this, patient eventually had a VATS procedure with pleural cavity exploration, decortication, and chest tube placement. Operative findings were consistent with entrapment of the lung with a surrounding thick peel. Chest tube was removed, but the PleurX catheter was kept in place. Although daily Chest X-rays showed stable moderate-sized pneumothorax, patient had clinical improvement and remained asymptomatic afterwards (Figure 5). Patient was eventually discharged with planned outpatient chemotherapy. 

## Discussion

Pneumothorax ex vacuo is a rare condition that occurs when air gets drawn into pleural space due to a negative intrapleural vacuum-like effect created from lung collapse. Development is usually seen in patients with bronchial obstruction from mucus plugs, aspirated foreing bodies, improperly positioned endotracheal tube and non-expanding lung occurring in the presence of fibrous peel from malignancy. Pathophysiology of pneumothorax ex vacuo is different when compared to primary or secondary pneumothorax. In pneumothorax ex vacuo, there is no breach in visceral pleural, rather a thick fibrous peel formation takes place due to neoplastic cells [2]. This phenomenon leads to lung entrapment and thus prevents reexpansion. Once the effusion is drained, due to the lung's inability to expand, we see a space that is called pneumothorax ex vacuo [3].

Pneumothorax ex vacuo as a complication was observed by Boland et al. in their analysis of patients with malignant pleural effusion requiring thoracentesis. Forty patients out of a total of 512 patients developed pneumothorax. Twenty-nine patients with pneumothoraces underwent catheter placement in the pleural space for treatment. Of these, 12 pneumothoraces were resolved and 17 remained unchanged. Of these 17 patients, no patient's lung was expanded post-insertion of a large bore chest tube although patients remained asymptomatic. Pleural effusion reaccumulated in all 17 after removal of the chest tube [4]. Analysis done by Heidecker et al. showed the presence of pneumothorax ex vacuo in 3% out of a total of 265 thoracocentesis [5]. Pneumothorax ex vacuo can present the predicament of treating vs observing. These pneumothoraces usually do not require treatment as they are likely from intra and extrapulmonary pressure equilibration [6]. 

Chest tube placement has not been shown to improve the condition significantly. However, simple observation has been practical, with spontaneous resolution observed over time [7]. In a case series study done by Kim et al., surgical intervention was done for a patient with PEV but that is usually rare and is a high-risk procedure. Spontaneous resolution was still deemed as the most likely outcome for PEV [8]. Pleural manometry can be a valuable tool to separate pneumothorax ex vacuo from other causes of non-expandable lungs. This can measure pleural pressures post chest tube clamping. In cases of pneumothorax ex vacuo, manometry will not show rise in pleural pressure over time. This helps distinguish from other traumatic pneumothorax, which can help guide management of an asymptomatic ex vacuo [9].

## Conclusions

Pneumothorax ex vacuo is a relatively rare complication that occurs due to lobar collapse from bronchial obstruction or due to entrapment of lung in malignant pleural effusion. Studies done previously show that treating pneumothorax ex vacuo with chest tube placement has shown no extra benefit. Simple observation is the best management plan. Although our case required VATS decortication post pneumothorax ex vacuo, it is important to know that surgical intervention has more risk than benefits. We suggest checking pleural manometry before thoracentesis, which can be very helpful in identifying or excluding the diagnosis of pneumothorax ex vacuo.
